# Attitudes and Skills in Basic Life Support after Two Types of Training: Traditional vs. Gamification, of Compulsory Secondary Education Students: A Simulation Study

**DOI:** 10.3390/pediatric16030053

**Published:** 2024-07-30

**Authors:** Adrián Rodríguez-García, Giovanna Ruiz-García, Rubén Navarro-Patón, Marcos Mecías-Calvo

**Affiliations:** 1 Departamento de Salud, Universidad Internacional Iberoamericana, Campeche 24560, Mexico; adrian.rodriguez@doctorado.unini.edu.mx; 2 Faculty of Health Sciences, Universidad Europea del Atlántico, 39011 Santander, Spain; 3 Institute of Secondary Education José del Campo, 39849 Ampuero, Spain; gruizg11@educantabria.es; 4 Faculty of Teacher Training, Universidade de Santiago de Compostela, 27001 Lugo, Spain; marcos.mecias@usc.es

**Keywords:** cardiopulmonary resuscitation, high school students, first aid, automatic external defibrillator, physical education

## Abstract

It is recommended to implement the teaching of Basic Life Support (BLS) in schools; however, studies on the best training method are limited and have been a priority in recent years. The objective of this study was to analyze the attitudes and practical skills learned during BLS training using a gamified proposal. A comparative study was carried out, consisting of Compulsory Secondary Education students [control group (CG; classical teaching) and experimental group (EG; gamified proposal)]. The instruments used were the CPR and AED action sequence observation sheet, data from the Laerdal Resusci Anne manikin and AED and Attitude Questionnaire towards Basic Life Support and the Use of the Automated External Defibrillator. Sixty-eight students (33 girls) with a mean age of 13.91 ± 0.70 years were recruited. Results were significantly better in the EG (n = 37) [i.e., breathing control (*p* = 0.037); call to emergency services (*p* = 0.049); mean compression depth (*p* = 0.001); self-confidence (*p* = 0.006); intention to perform BLS and AED (*p* = 0.002)]; and significantly better in the CG (n = 31) [Total percentage of CPR (*p* < 0.001); percentage of correct compression (*p* < 0.001); time to apply effective shock with AED (*p* < 0.001); demotivation (*p* = 0.005). We can conclude that the group that was trained with the training method through the gamified proposal presents better intentions and attitudes to act in the event of cardiac arrest than those of the classic method. This training method allows for similar results in terms of CPR and AED skills to classical teaching, so it should be taken into account as a method for teaching BLS to secondary education students.

## 1. Introduction

International institutions such as the European Resuscitation Council (ERC) enacted the “Kids Save Lives” initiative in 2015, which recommended including CPR in the school curriculum worldwide and promoting CPR training for schoolchildren aged 12 years or older [1]. Little by little, different countries and regions of the world joined this initiative and introduced the teaching of first aid and basic life support (BLS) into their study plans as necessary content [2,3]. In December 2020, the Spanish government took another step in this regard with the approval of Ley Orgánica 3/2020, de 29 de diciembre, por la que se modifica la Ley Orgánica 2/2006, de 3 de mayo, de Educación (LOMLOE) [4]. This law included contents such as first aid, the lateral safety position, or the conduct of Protect, Alert, and Help (PAH) within the minimum teachings of Compulsory Secondary Education (Real Decreto 217/2022, de 29 de marzo, por el que se establece la ordenación y las enseñanzas mínimas de la Educación Secundaria Obligatoria) [5] in subjects such as Physical Education.

Despite the recommendation to include BLS instruction in schools, little is known about the best methods to enable and implement it [6]. In recent years, international guidelines have been simplified to improve skills in performing CPR and using AEDs [7]. Teaching methods have varied since the teaching of first aid and BLS began until today [8]. Thus, we have gone from traditional teaching and practical skills of about 50 min, considered the “gold standard”, to today, where there are numerous training methods ranging from mass training using small training pills [9] to the so-called active methodologies, among which are educational “scape rooms” [10], “augmented reality” [11], or “gamification” to teach BLS, although the latter cases have been little studied, and current evidence does not clearly demonstrate a consistent benefit compared to other BLS training methods [12].

Although recommendations on standardized teaching and assessment methods have been noted to be necessary to understand the best ways to train children in theoretical knowledge and practical skills in BLS [13], little is known about which of these motivational training methods. It is known that training in knowledge and skills for performing CPR and the use of AEDs is no guarantee that trained personnel will show a positive attitude to perform CPR in a real situation [14,15]. This attitude is influenced by factors such as the belief of insufficient capacity [15]; fear of transmitting diseases or causing injuries [16].

Therefore, BLS and AED training methods should focus on teaching theoretical knowledge, practical skills and promoting appropriate attitudes to perform CPR and apply defibrillation with an AED if necessary, thereby contributing to improved survival after cardiac arrest [17].

More studies are needed to know what types of training have a similar effect on skill learning and the steps to follow to identify cardiac arrest, perform quality CPR, as well as be able to perform effective defibrillation. In addition, it is also necessary to evaluate the intention, attitude, and self-confidence to apply CPR and use the AED. In this sense, the hypotheses of this study are: (1) a gamified teaching–learning program is just as feasible and effective as a traditional teaching–learning process for learning to monitor the BLS action sequence, the quality of external cardiopulmonary resuscitation, and perform an effective shock; and (2) a gamified teaching–learning proposal increases the intention, attitude, and self-confidence of schoolchildren to perform CPR and use the AED.

For all of the above, the objective of this study was to evaluate the feasibility and effectiveness of a teaching–learning process, through a gamified proposal on BLS and the application of an effective shock with an AED, as well as to evaluate the intention, attitude, and self-confidence of schoolchildren to perform CPR and use the AED.

## 2. Materials and Methods

### 2.1. Study Design

For this quasi-experimental study with a control group, 77 students who were studying Compulsory Secondary Education at an educational center in Cantabria (Spain) were invited to participate. They completed a course taught through a traditional/classical class and practical BLS-AED skills, according to the recommendations of the European Resuscitation Council [18], and another following the same recommendations, but using a gamified approach. The inclusion criteria to participate in the study were: (1) have the signed informed consent of their parents or legal guardians; (2) do not suffer from any illness that prevents them from answering the questionnaires and completing the training; and (3) do not have a BLS-AED training diploma accredited in the 3 previous years or repeat the training.

### 2.2. Participants

The study population was made up of two groups of secondary education students: a first group (CG; n = 31) that completed a course through traditional/classical BLS-AED teaching and a second group (EG; n = 37) that completed a BLS-AED course through a gamified proposal. In both groups, those students who had an accredited BLS-AED training diploma within the previous 3 years, or who were repeating the training [i.e., CG (n = 5); EG (n = 4)], were discarded in the data analysis.

### 2.3. Instruments, Materials, and Measurements

Firstly, sociodemographic data were collected from the participants: age, sex (boy/girl), and previous training with a degree (yes/no).

#### 2.3.1. Hands-Only CPR Data Collection

The CPR manikin employed in performance and data collection was the Laerdal Resusci Anne manikin, with Skill reporter software version 2.4 (Laerdal medical AS, Stavanger, Norway), set for chest compressions only mode.

*BLS action sequence:* the BLS actions were assessed using an ad hoc checklist that reported if performed or not each single item including on-scene safety, consciousness assessment and breathing (forehead–chin maneuver and see–hear–feel), call to emergency services, and starting external chest compressions. Observers recorded whether the step was executed (Yes/No).

*Quality of External Cardiopulmonary Resuscitation:* This was carried out with the Laerdal Resusci Anne manikin, measuring compression quality parameters as established under ERC 2021 standards [18]: compression depth of 50 to 60 mm; compression rate of 100 to 120 compressions/minute. The variables recorded were: mean compression depth (5–6 cm); correct re-expansion percentage (>75%); correct compression percentage (>75%); total number of compressions in 2 min (200–240); and overall quality of CPR (>75%).

#### 2.3.2. AED Data Collection

The source of instruction for the handling of the AED was the Laerdal Training AED, a simulator of the Heartstart FR2+ Phillips defibrillator. The collected variables were as follows: (1) effective discharge (effective shock), (2) safety in execution, (3) quality target, (4) error made, (5) time to make an effective discharge.

Shock was considered effective if the shock was performed without any of the errors, which varied the target of the shock, thoracic patch below mid-line of the heart, costal patch below mid-line of the heart, performing the shock but without placing the patches, performing the download without gluing the patches or incorrect placing. Safety in execution was considered safe if the participant was not in contact with the manikin at the time of the applying the shock.

#### 2.3.3. Attitude towards Basic Life Support and Automated External Defibrillator Use Questionnaire

This questionnaire was used to check the intention and attitudes towards carrying out BLS [14], once the training programs were applied, both in the CG and the EG. This questionnaire is composed of 16 items on a Likert-type scale (from 1 to 7; where 1 means “I do not agree at all”, 4 means “neutral”, 7 means “totally agree”). The scale consists of the following four factors: self-confidence (for example: “because physical education is fun”); intention to perform BLS and AED (for example: “If I were the only witness of cardiac arrest in an unknown person, I would start resuscitating him”); demotivation (“I would not do resuscitation because I would be afraid of the possible consequences (rib fracture, complaint, etc.”); positive motivation (“Knowledge of BLS with AED is a useful skill for everyday life”).

#### 2.3.4. Training Programs

The two training programs used (traditional vs. gamified approach) were designed in accordance with current international BLS guidelines [18]. Furthermore, the practical part of the skills was the same in both training experiences, so that each participant practiced a total of 6 min in 2 min shifts of continuous compressions (after the sequence of cardiac arrest detection actions), and the use of the AED following the instructions when they did not perform the 2 min of external cardiac massage [19].

The traditional training program was carried out following the guidelines of the European Resuscitation Council [18], so the students received “a 40-min theoretical-practical course, with instructor-led training in BLS and AED in groups of 10 people, during which the importance of performing external cardiac compressions without interruptions was emphasized” [19]. In practical skills work on external cardiac compressions and AED use, participants performed the sequence of actions to identify cardiac arrest (safety scene; consciousness assessment; breathing assessment; emergency call), external cardiac compressions on a manikin with immediate feedback, allowing participants to compress and re-expand the manikin’s chest and follow the appropriate compression ratio, exchanging compressions every 2 min, and applying the AED to the chest 3 times on the manikin’s chest to deliver a shock.

**Figure 1:**
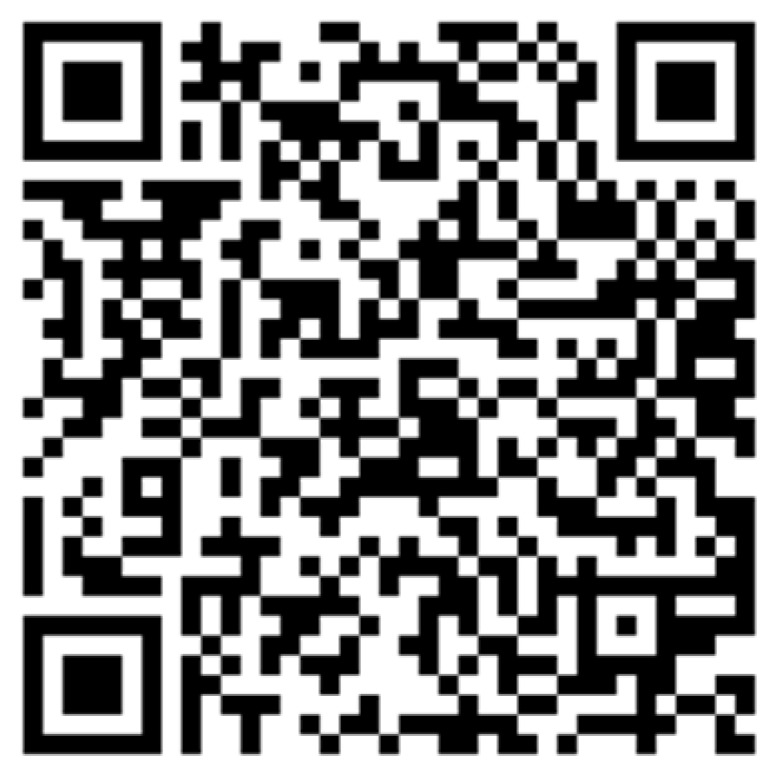
QR code access to train through the gamified proposal.

The training program using the gamified proposal was designed following the same instructions as the traditional one [18]. For the development of the theoretical contents, these were adapted with the theme of Survivors, where the students had to overcome various missions to save their teacher, who was injured and needed their help. The presentation can be accessed through (Figure 1).

**Figure 2:**
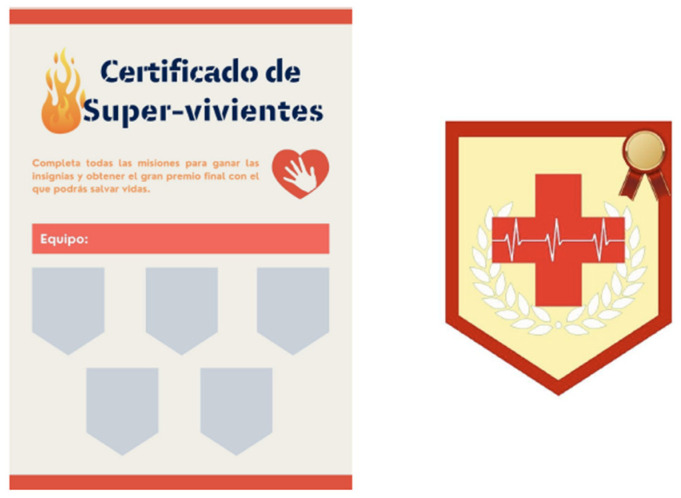
Survivor certificate and badge model given to the group each time a proposed challenge is overcome.

This presentation was used to put the students in the situation. Five teams were created in each class, with approximately 6 components each, with a different component from each group having to perform in each challenge, and all at the same time in the group tests. When a group or several solved a challenge well, they were awarded a badge that they had to stick on their survivor certificate (Figure 2).

The challenges were the following (Figure 1):

Challenge 1: Introductory video and PAH

Challenge 2: Airway

Challenge 3: Circulation

Challenge 4: CPR

Challenge 5: AED use

This process had a duration similar to the classic/traditional methodology (50 min). For the practical part, the same procedure and schedule were used as for the traditional teaching group.

### 2.4. Procedure

The school management and physical education teachers were contacted, and the purpose of the study was explained to them. Once approval was received, an informed consent form was sent to the parents, where they were provided with all the necessary information about the study, and where their children’s voluntary participation was explained, and they could withdraw them at the time that they consider appropriate. Before providing the training, the personal data of the students who agreed to participate (age, sex, and knowledge or previous training accredited in BLS) were collected. The training method established for it was applied to each group (CG: traditional method; EG: training through a gamified proposal), in the Physical Education class schedule of each group (in our country, these classes last 50 min): first the theoretical part, and then the practical skills part. After training, students were tested on the sequence of actions to detect possible cardiac arrest; on the quality of external cardiac compressions; on the results obtained and the mean time in applying an effective discharge. Once the teaching–learning program (traditional–gamified) was applied, each participant, individually, went to an isolated room in the school itself and was explained that they were in the following situation: “Imagine that this mannequin is a person who has collapsed in front of you, and you are the only person who knows how to help him. Do what you think should be done in these situations”. The Attitude Questionnaire towards Basic Life Support and the Use of the Automatic External Defibrillator was also administered.

### 2.5. Statistical Analysis

SPSS software (SPSS v.25, IBM Corporation, New York, NY, USA) was used for all statistical analyses. The level of significance was set at *p* < 0.05.

Means and standard deviation were used to express the central tendency of the quantitative data, while frequencies and percentages were used to express the categorical variables. After the application of the training procedure in both groups, on the one hand, Pearson’s χ^2^ test was used to evaluate the differences between the groups (CG vs. EG), in terms of the action sequence prior to the application of external cardiac compressions. For the application of the AED, the same statistical test was carried out regarding effective discharge, security in execution, or achievement of the quality objective. On the other hand, a multivariate analysis (MANOVA) was carried out for each variable studied in relation to the quality of hands-only CPR and the time necessary to apply an effective shock with the AED, as well as those of the questionnaire (self-confidence; intention to perform BLS and AED; demotivation; positive motivation). Group (CG vs. EG) and sex (man vs. woman) were included as factors. For the main effects and interactions, the Bonferroni statistic was implemented, and the eta squared (η^2^) metric of statistical power was used. Association was considered weak with values between 0.10 and 0.29, moderate if the value ranged between 0.30 and 0.49, and strong for values ranging between 0.50 and 1.00. Cronbach’s alpha coefficient was subsequently used to assess the reliability of different subscales.

### 2.6. Ethical Considerations

Ethical approval was obtained by the Ethics Committee of the Universidad de Internacional Iberoamericana with code number CR-222. During their participation in this research, subjects were treated with respect to the Declaration of Helsinki. The study was conducted in accordance with international research standards, including the guidelines of the Publication Manual of the American Psychological Association regarding the appropriate treatment of participants and ensuring necessary care during the study [20].

## 3. Results

A total of 68 students participated in the research and were divided into two groups, CG (n = 31; 14 girls) and EG (n = 37; 18 girls), with a mean age of 13.91 ± 0.70. The results obtained in each of the dimensions that make up the study are reported below.

### 3.1. CPR Results

#### 3.1.1. Action Sequence

Table 1 shows that, in all the items evaluated, there are statistically significant differences in favor of the EG in respiratory control (*X*^2^ = 4.333; *p* = 0.037; Cramer’s V = 0.252; *p* = 0.037), and in the call to services of emergencies (*X*^2^ = 3.882; *p* = 0.049; Cramer’s V = 0.239; *p* = 0.049), but not in the safety of the scene (*p* = 0.899) or in the verification of consciousness (*p* = 0.623).

#### 3.1.2. Hands-Only CPR Quality

In Table 2, it can be seen that the means of the global scores of the CG and EG were similar, with the CCP and the CRP, TCN and OcprP being within the established ranges in the CG, both in boys and in girls; in the EG the MCD, the CCP in boys, and the CRP in both boys and girls.

The findings from the analysis of the results on the MCD variable show that the training method factor has a significant main effect [F(1, 64) = 13.043, *p* = 0.001; η^2^ = 0.169], since the CG reaches a greater mean depth, but it is the EG that does so in the appropriate range (50–60 mm). The sex factor also has a significant main effect on this variable [F(1, 64) = 6.723, *p* = 0.012; η^2^ = 0.095]. Boys (63.57 ± 10.93) reach a greater mean depth, but it is girls who are in the appropriate range (56.57 ± 13.06). No statistically significant differences were found in the interaction between both factors in this variable (*p* = 0.835). When making pairwise comparisons, statistically significant differences were found between boys in the CG (69.18 ± 7.85) and those in the EG (58.84 ± 11.08) (*p* = 0.008), as well as between girls in the CG (61.60 ± 2.87) and those of the EG (52.38 ± 2.62) (*p* = 0.021).

When the results of the CCP are analyzed, it is observed that there is a significant effect on the group factor [F(1, 64) = 8.914, *p* = 0.004; η^2^ = 0.122], being better in the CG participants than in the EG participants. No statistically significant differences were found either in the sex factor (*p* = 0.062) or in the interaction between both factors (*p* = 0.642). In pairwise comparisons, statistically significant differences were found between girls in the CG (86.06 ± 18.60) and girls in the EG (58.50 ± 45.65) (*p* = 0.019).

The results regarding CRP indicate that there is only a significant effect on the sex factor [F(1, 64) = 4.034, *p* = 0.049; η^2^ = 0.059], being better in girls (94.54 ± 17.28) than in boys (83.14 ± 27.06), although in both cases they are above the adequate percentage (>70%). Statistically significant differences were also found between boys (76.57 ± 33.87) and girls (92.22 ± 22.43) of the EG (*p* = 0.039).

In the analysis of the OcprP variable, there is a significant main effect in the group factor [F(1, 64) = 20.314, *p* < 0.001; η^2^ = 0.241]. There is no significant main effect either in the gender factor (*p* = 208) or in the interaction between both factors (*p* = 0.990). In pairwise comparisons, statistically significant differences were found between boys in the CG (93.06 ± 6.58) and those in the EG (65.15 ± 28.29) (*p* = 0.002), and between girls in the CG (85.13 ± 17.66) and those of the EG (57.38 ± 35.70) (*p* = 0.003).

In the rest of the variables analyzed, no statistically significant differences were found between the factors group, sex, or the interaction between them (*p* > 0.050).

### 3.2. AED Results

#### 3.2.1. AED Application

A total of 30 participants (96.8%) from the CG and 37 (100%) from the EG (Table 3) simulated an effective shock (*p* = 0.271), without making any error that would prevent it (e.g., chest patch below the midline of the heart; costal patch below the midline of the heart; perform the shock without applying the patches without gluing the patches or placing them incorrectly). On the other hand, 28 participants (90.3%) of the CG and 37 participants (100%) of the EG managed to apply a safe shock (that is, without touching the manikin while the shock is applied) (*p* = 0.053). Finally, of the 68 participants who achieved the objective, 9 participants (29.0%) from the CG and 11 (29.7%) from the EG, also achieved the quality objective, that is, they did not make any errors, they did it safely and the order of execution was correct (*p* = 0.950).

#### 3.2.2. Mean Times to Apply an Effective Shock with the AED

Table 4 shows the mean times (in seconds) of those participants who managed to perform an effective shock (i.e., CG = 29; EG = 37). The results of the MANOVA indicated that there are statistical differences in the training method factor [F(1, 64) = 67.426; *p* < 0.001; η^2^ = 0.513], but not in the sex factor (*p* = 0.157), nor in the interaction of both factors (*p* = 0.368), with the CG schoolchildren spending the least time giving an effective shock. In pairwise comparisons, statistically significant differences were found between boys in the CG (*p* < 0.001) and those in the EG and between girls in the CG (*p* < 0.001) and those in the EG.

### 3.3. Questionnaire Results

Table 5 shows the descriptive statistics of the variables used, the reliability analysis, and their correlation. The results of the reliability analysis showed adequate values in self-confidence (SC), intention to perform BLS and AED (ItP), demotivation (Dm), and positive motivation (PM). In relation to the correlation analysis, the high and positive correlation between SC and ItP and MP stands out, as does the negative relationship between Dm and SC, ItP and MP. The positive and significant relationship between ItP and MP also stands out.

The results regarding the Attitude Towards Basic Life Support and Automated External Defibrillator Use Questionnaire can be seen in Table 6. It is observed that the scores given by the EG participants are higher than those of the CG in SC, ItP, and PM. On the contrary, participants in the CG have higher values in Dm than those in the EG.

The results of the analysis carried out indicate that there is a significant main effect of the group factor on SC [F(1, 64) = 8.132; *p* = 0.006; η^2^ = 0.113] and ItP [F(1, 64) = 10.928; *p* = 0.002; η^2^ = 0.146], with the mean scores higher in the EG than in the CG. Regarding Dm, there is a significant main effect on the group factor [F(1, 64) = 18.015; *p* < 0.001; η^2^ = 0.220], but with higher mean scores in the CG than in the EG. No statistically significant differences were found in the sex factor (*p* > 0.05) or in their interactions (*p* > 0.05). No effect was found on PM (*p* > 0.05).

## 4. Discussion

This quasi-experimental study aimed to compare the results of two teaching–learning processes in BLS, one using a traditional method (CG) versus another that used a gamified proposal (EG), and its impact on the monitoring of the action sequence to detect cardiac arrest, the skills to perform hands-only CPR, as well as the procedure for using the AED and delivering an effective shock, and the attitude and intention to perform CPR and use the AED. Based on the overall results obtained, it can be said that training students through a gamified proposal is another of the training methods that can be just as effective as those used so far [8]. Both training programs used in this study (traditional and gamified) allow secondary school students to be able to follow the previous steps (safety of the scene, assessment of consciousness and breathing, and call to emergency services) to identify a cardiac arrest and begin applying external cardiac compressions appropriately (depth, percentage of compression, correct re-expansion of the chest, and number of appropriate compressions). These results agree with those found in previous research using the so-called active methodologies in primary and secondary education students [21,22]. This type of methodology allows the student to deliver an effective shock with the AED by following the proper steps or making minimal errors (turning on, sticking electrodes on the patient’s bare chest, plugging in the connector, and following the instructions). Therefore, we must accept the first hypothesis, which stated that a gamified teaching–learning program is just as feasible and effective as a traditional teaching–learning process for learning to monitor the BLS action sequence, the quality of external cardiopulmonary resuscitation, and perform an effective shock.

Finally, the program with the gamified proposal causes students who received training using this method to have higher scores in intention to perform BLS and use of the AED, in SC and PM than those who received the traditional training. These three discussed aspects were identified as key for laypeople to provide BLS in an out-of-hospital cardiac arrest event [14,15,16,23,24]. Due to these results, we must accept hypothesis 2, which states that a gamified teaching–learning proposal increases the intention, attitude, and self-confidence of schoolchildren to perform CPR and use the AED.

Specifically, the EG obtained better results in checking breathing and calling emergency services, but not in the safety of the scene or in checking consciousness, results similar to previous studies [15,16,17,22,24,25]. If we take into account that the steps to follow are difficult to learn and follow [25], even for trained adults [26], it is striking in this study that more than half of the participants (CG and EG) were able to follow the sequence to identify cardiac arrest and begin external cardiac compressions [27]. This has happened except in the call to the emergency service, which could have been due to the false security provided by being in the school itself and thus avoiding this step [28].

Regarding the quality values of external cardiac compressions [18], the EG students reached an MCD, within the appropriate values (50–60 mm), while the CG students exceeded this range (>60 mm), an aspect that is usually difficult to achieve [29]. Furthermore, and relating this variable to correct thoracic re-expansion, boys were the ones who reached a greater mean depth compared to girls, and girls were the ones who presented the highest percentage of thoracic re-expansion [22,30,31], and this may be due to anthropometric aspects such as the height or weight of the participants [32,33].

Contrary to what would be expected in the CCP, as it is related to the mean depth of the compressions, it is the CG that achieves the best percentage in this variable of external chest compressions, although in the EG this percentage also reaches 70% established as quality in this study [34]. Regarding the TCN in 2 min, participants in both training programs presented an adequate number, without significant differences. This could be due to the real-time feedback provided by the manikin during training [35]. Finally, the OcprP was significantly better in the CG, since it is related to all the previous parameters [18,36], which may be due to the fact that they also present better percentages in compression and re-expansion of the chest [37].

The percentages in terms of achieving the quality objective in the use of the AED were low in both groups, as was already reported in other studies [21,22,26,38]. However, almost 100% of the participants in both groups were able to apply an effective shock in just over 1 min [22], and with a similar percentage when applying it without being in contact with the manikin (security). The results of this study are superior to those achieved by active or training teachers [9,19].

The results of the analysis of the variables of the Attitude Questionnaire towards Basic Life Support and the Use of the Automatic External Defibrillator highlighted the positive correlation between SC, ItP, and PM, as well as the negative relationship between Dm and the SC, ItP and PM [39,40]. The above can give us an idea of the importance of making an effort on the part of educators to motivate schoolchildren to carry out BLS and AED in the training [14,41] or carry out periodic training to increase SC [40]. Despite the evidence and the importance of motivation towards the BLS, in our study there were no differences between the groups in the PM variable, which gives us an idea that these contents, due to their little treatment in the classrooms, are novel and motivating regardless of the strategy used for teaching. On the other hand, students who received training through the gamified proposal showed greater SC, ItP, and PM than those in the CG group [22]. This may be because active teaching, through the challenges of the gamified proposal and the cooperation necessary for its achievement and improvement, has better performance since the student feels more motivated, participates in the learning process in an immersive way, and is more interested [42].

Regarding self-confidence, it could be reinforced with individual practical training on manikins, with which schoolchildren observed their skills and abilities, and therefore, improved their self-confidence [15,43]. For this reason, special emphasis should also be placed on this during the BLS and AED courses for schoolchildren, since through gamified proposals adapted to the level of understanding and development of the participants, they are being offered a better self-perception towards the application of the BLS and the AED [14]. Therefore, it would be contributing to reducing the most commonly reported reasons for not performing CPR [15,16,23,24].

Regarding the limitations of the study, it is necessary to highlight a small sample of students, which, although it could be considered non-representative, is a first step for the assessment of this training method, both in terms of knowledge, skills, and attitudes, compared to the CPR and use of the AED. On the other hand, we must emphasize that this has been a simulation study, so it is unknown how the participants would act if it were a real situation, both at an attitudinal and procedural level. Finally, no medium–long-term follow-up has been carried out (degree of forgetting), so we only know the results after the training.

## 5. Conclusions

The results of this study indicate that a BLS training program, using the proposed gamification, ensures that participants learn to properly follow the sequence of actions to detect cardiac arrest and initiate and perform external cardiac compressions, according to the recommendations of international organizations. Likewise, the training program based on the gamified proposal ensures that 100% of the participants are able to perform an effective download and do so in just over 1 min, just like the group that received classical/traditional teaching. Finally, we can conclude by indicating that the training program using the gamified proposal allows us to obtain similar results in terms of knowledge and skills, both in CPR and in the application of a shock with the AED, but it generates greater positive motivation, greater perceived self-efficacy, and greater intention to perform CPR and use the AED than the traditional program and, at the same time, less demotivation.

## Figures and Tables

**Table 1:** Results of the descriptive analysis carried out in the action sequence to detect possible cardiac arrest.

		CG (n = 31)	EG (n = 37)
Safety scene	No	1 (3.2%)	1 (2.7%)
	Yes	30 (96.8%)	36 (97.3%)
Consciousness assessment	No	6 (19.4%)	9 (24.3%)
	Yes	25 (80.6%)	28 (75.7%)
Breathing assessment	No	7 (22.6%)	2 (5.4%)
	Yes	24 (77.4%)	35 (94.6%)
Emergency call	No	20 (64.5%)	15 (40.5%)
	Yes	11 (35.5%)	22 (59.5%)

**Table 2:** Descriptive data of the analyzed variables (mean, standard deviation) according to sex and time after training in the quality of hands-only CPR.

		CG		EG	
Variable		M	SD	M	SD
MCD (mm)	Boys	69.18	7.85	58.84	11.07
	Girls	61.60	8.17	52.38	15.02
	Total	65.51	8.77	55.70	13.36
CCP (%)	Boys	96.06	6,84	76.68	35.64
	Girls	86.06	18.60	58.50	45.65
	Total	90.74	14.70	67.83	41.28
CRP (%)	Boys	90.93	12.80	76.57	33.87
	Girls	97.33	7.52	92.22	22.43
	Total	94.03	10.90	84.18	29.56
TCN (2 min)	Boys	234.31	51.34	227.26	51.34
	Girls	240.86	42.10	222.44	42.10
	Total	237.48	46.49	224.91	46.49
OcprP	Boys	93.06	6.58	65.15	28.29
	Girls	85.13	17.66	57.38	35.70
	Total	89.22	13.54	61.37	31.90

Note: M: Mean; SD: Standard Deviation; MCD: mean compression depth; CCP: correct compressions percentage; CRP: correct re-expansion percentage; TCN: total compressions number; OcprP: Overall cardiopulmonary resuscitation percentage.

**Table 3:** Results of the descriptive analysis of the variables analyzed on the AED.

		CG (n = 31)	EG (n = 37)
Objective surpassed	No	1 (3.2%)	0 (0.0%)
	Yes	30 (96.8%)	37 (100%)
Security in execution	No	2 (9.7%)	0 (0.0%)
	Yes	28 (90.3%)	37 (100%)
Quality objective	No	21 (71.0%)	26 (70.3%)
	Yes	9 (29.0%)	11 (29.7%)

**Table 4:** Descriptive statistics of the time variable according to sex and group.

Variable		Boys		Girls		Total	
	Group	Mean	SD	Mean	SD	Mean	SD
AED application time	CG	34.12	7.31	46.13	11.66	39.93	11.28
	EG	80.92	21.23	82.62	32.17	82.24	26.76

Note: SD: Standard Deviation.

**Table 5:** Results of the Attitude Towards Basic Life Support and Automated External Defibrillator Use Questionnaire.

Dimensions	M	SD	A	K	α	SC	ItP	AM	MP
Self-confidence (SC)	5.67	0.93	−0.62	0.19	0.720	1	0.690 **	−0.386 **	0.614 **
Intention to perform (ItP)	6.21	0.90	−1.07	0.29	0.795	1	1	−0.462 **	0.495 **
Demotivation (Dm)	3,05	1.43	−1.38	1.43	0.797	-	-	1	−0.374 **
Positive Motivation (PM)	6.13	1.04	0.38	−0.19	0.739	-	-	-	1

Note: M = Mean; SD = Standard Deviation; A = Asymmetry; K = Kurtosis; α = Cronbach’s Alpha; ** The correlation is significant at the 0.01 level (Bilateral).

**Table 6:** Descriptive data according to sex and group of the Attitude Questionnaire towards Basic Life Support and the Use of the Automatic External Defibrillator variables.

		CG		EG	
Variable		M	SD	M	SD
Self-confidence (SC)	Boys	5.21	0.88	5.85	0.98
	Girls	5.47	0.90	6.06	0.75
	Total	5.34	0.88	5.95	0.87
Intention to perform (ItP)	Boys	5.58	1.06	6.49	0.52
	Girls	6.06	0.93	6.51	0.81
	Total	5.81	1.01	6.50	0.67
Demotivation (Dm)	Boys	3.81	1.25	2.52	1.15
	Girls	3.77	1.30	2.35	1.50
	Total	3.79	1.25	2.44	1.32
Positive Motivation (PM)	Boys	5.89	0.94	6.22	1.37
	Girls	5.82	0.93	6.42	0.83
	Total	5.86	0.92	6.32	1.12

Note: M = Mean; SD = Standard Deviation.

## Data Availability

The data presented in this study are not available in accordance with Regulation (EU) of the European Parliament and of the Council 2016/679 of 27 April 2016 regarding the protection of natural persons with regard to the processing of personal data and the free circulation of these data (RGPD).
